# Characterization of Sn–Sb–Ti Solder Alloy and the Study of Its Use for the Ultrasonic Soldering Process of SiC Ceramics with a Cu–SiC Metal–Ceramic Composite

**DOI:** 10.3390/ma14216369

**Published:** 2021-10-25

**Authors:** Roman Kolenak, Igor Kostolny, Jaromir Drapala, Paulina Babincova, Matej Pasak

**Affiliations:** 1 Faculty of Materials Science and Technology in Trnava, Slovak University of Technology in Bratislava, Jána Bottu č. 2781/25, 917 24 Trnava, Slovakia; roman.kolenak@stuba.sk (R.K.); paulina.babincova@stuba.sk (P.B.); matej.pasak@stuba.sk (M.P.); 2 FMT—Faculty of Materials Science and Technology, Technical University of Ostrava, 17. Listopadu 15, 708 33 Ostrava, Czech Republic; jaromir.drapala@vsb.cz

**Keywords:** ultrasonic, intermetallic compounds, solder, composite

## Abstract

The aim of this research was to characterize soldering alloys of the type Sn–Sb–Ti and to study the ultrasonic soldering of SiC ceramics with a metal–ceramic composite of the type Cu–SiC. The Sn5Sb3Ti solder exerts a thermal transformation of a peritectic character with an approximate melting point of 234 °C and a narrow melting interval. The solder microstructure consists of a tin matrix, where the acicular constituents of the Ti_6_(Sb,Sn)_5_ phase and the sharp-edged constituents of the TiSbSn phase are precipitated. The tensile strength of the soldering alloy depends on the Ti content and reaches values from 34 to 51 MPa. The average strength of the solder increases with increasing Ti content. The bond with SiC ceramics is formed owing to the interaction of titanium, activated by ultrasound, with SiC ceramics, forming the (Ti,Si)_6_(Sb,Sn)_5_ reaction product. The bond with the metal–ceramic composite Cu–SiC is formed owing to the solubility of Cu in a tin solder forming two phases: the wettable η-Cu_6_Sn_5_ phase, formed in contact with the solder, and the non-wettable ε-Cu_3_Sn phase, formed in contact with the copper composite. The average shear strength of the combined joint of SiC/Cu–SiC fabricated using the Sn5Sb3Ti solder was 42.5 MPa. The Sn–Sb–Ti solder is a direct competitor of the S-Bond active solder. The production of solders is cheaper, and the presence of antimony increases their strength. In addition, the application temperature range is wider.

## 1. Introduction

The direct, fluxless soldering of combinations of metallic, non-metallic, or composite materials offers great advantages from both technological and economical viewpoints. It is not necessary to deposit the coatings on hard-to-solder surfaces, nor to apply special interlayers to ensure the wettability of substrates with solders. These technological and economic priorities of production of heavy-duty electronic devices are the driving force of modern times. At the same time, it is necessary that these devices operate faster, more reliably, and economically. The core of these devices consists of heavy-duty transistor semiconductor parts, which in one package create a powerful electronic chip [1,2,3]. As a primary semiconductor material, silicon carbide (SiC) has become ever more popular nowadays. Its use results in breakthrough performance, smaller dimensions, and lower power consumption [4,5,6].

The most frequent type of failure of power modules involves thermal fatigue of soldered joints owing to the different coefficients of thermal expansion (CTE) of semiconductor chips and packaging materials. Therefore, for thermal coolers or heat sinks, materials with reduced coefficients of thermal expansion in combination with high thermal conductivity are required [7,8]. Due this reason, the Cu–SiC composite, combining high thermal conductivity with low coefficient of thermal expansion, has begun to be used in present times [9].

Direct soldering of ceramics to Cu–SiC brings about a number of issues to be solved in order to attain a sound joint. One of the most essential issues consists in the non-wetting of ceramic materials on use of commercial solders. This issue may be solved by use of active solders containing elements with a high affinity to some components of ceramics. Then, it is unnecessary to coat the ceramic materials with solderable coatings. Titanium is the most frequently used element that is added to soldering alloys, but other active metals are also used. The performance of solders alloyed with titanium is documented in many studies [10,11,12,13,14,15,16,17]. Titanium allows wetting and soldering of Ti, Al, Si, glass, and different types of ceramics owing to the activation of soldered surfaces [18,19]. Sn–Ag–Ti is the most used base of an active solder with Ti addition, initially developed by S-Bond Technologies. This base joins most metals, ceramics, and composites. It is applied without flux or preliminary coating of the substrates, and it does not contain lead or cadmium, thus conforming to requirements of all initiatives regarding lead-free soldering (RoHS, etc.). The solderability of metallic or ceramic materials by Sn–Ag–Ti-based solders is relatively well documented in the literature [20,21,22,23,24,25,26]. The authors of these publications have proved the suitability of the base for soldering of ZnS–SiO_2_, ITO, Al_2_O_3_, MAO-coated Al alloy, TiO_2_, graphite, and AlN. Titanium in a solder ensures the formation of a reaction zone in the solder/substrate boundary and thus the wetting of these materials. The development of active solders is in progress, and new solder bases that would be capable of competing with the S-Bond active solders are being designed. In this study, the Sn–Sb–Ti solder base, on which the patent protection under the number WO2020115653 (A1)-2020-06-11 is valid [27], were used. Although the Sn–Sb–Ti base was studied by Berger et al. [28], only the ternary diagram of this base was investigated. This solder is a direct competitor to the S-Bond active solder and as the soldering alloy, it will be published for the first time in this work. The production of a solder is cheaper, and the presence of antimony increases its strength. In addition, the application temperature range is wider.

The direct soldering of SiC/CuSiC combination by use of the Sn–Sb–Ti solder similarly requires the selection of the correct technology since it is necessary to activate the Ti element in the solder. Activation at high temperatures is impossible since the metal component of the composite would be molten. Application of ultrasonic soldering for the formation of bonds between CuSiC and SiC seems to be one of the most suitable methods applicable for fluxless soldering of such materials. This process is highly productive. The time necessary for bond formation is just on the order of several minutes. The soldering temperature is 20–30 °C above the melting point of the solder, and the activation of the active element is ensured by the application of ultrasound. The suitability of this method has been approved by many professional publications from the field of brazing and soldering [29,30,31,32] and the field of transient liquid phase (TLP) bonding [33,34,35,36,37].

The aim of the present study was the analysis of the new active solder based on Sn–Sb–Ti and soldering of SiC ceramics with a metal–ceramic composite of the type CuSiC. Soldering was performed by a fluxless process with the application of ultrasound. The interactions among the solder, ceramic, and composite substrates were investigated. The shear strength of fabricated joints was measured, and their fractured surfaces were analyzed.

## 2. Materials and Methods

After determination of weight proportions of the prepared alloy, the weighing of individual components was performed. As the input components for solder manufacture, the materials with a high purity of 4*N* were used.

The solder manufacture in the as-cast condition was realized in a vacuum oven. The procedure was as follows: All weighed metals were put into a crucible made of Al_2_O_3_ ceramic. The induction vacuum oven with evacuated air atmosphere was applied. The experiment was performed in an argon overpressure of 200 Mbar. The gas Argon 4.6 was applied. The temperature of the solder manufacture was around 1100 °C. Titanium was slowly dissolved in the solder.

The experimental chemical composition is given in Table 1. Chemical analysis was performed using atomic emission spectrometry with induction-coupled plasma (ICP-AES). The analysis was realized on the equipment SPECTRO VISION EOP. The specimens for ICP-AES analysis were dissolved in suitable chemical solutions of acids and bases. Proper analysis was performed on the atomic emission spectrometer with a pneumatic atomizer and Scott’s sputtering chamber.

**Figure 1:**
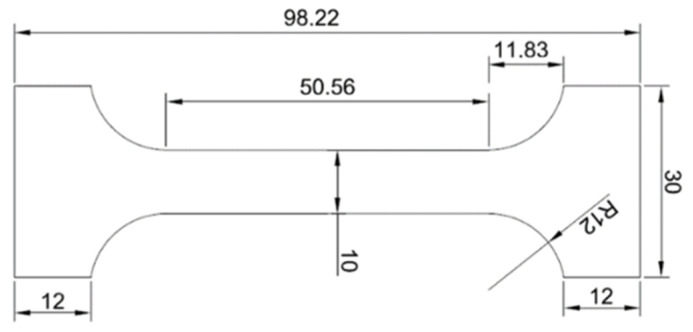
Test piece of a solder for a static tensile test.

Then a test piece for tensile strength test was machined from the soldering alloy (Figure 1). The dimensions in Figure 1 are given in millimeters.

**Figure 2:**
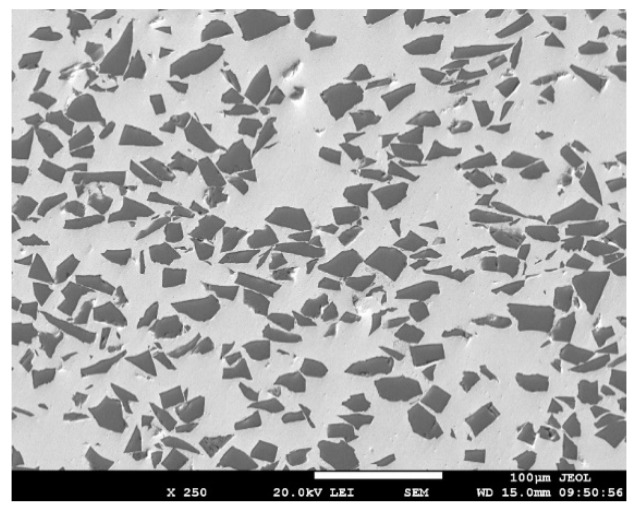
Microstructure of a metal–ceramic composite of the type Cu–SiC.

The following materials were used as substrates in the experiments:

- A ceramic substrate of SiC in the form of discs Ø 15 × 3 mm and for a shear strength test in the form of squares with dimensions of 10 × 10 × 3 mm.
- A composite substrate composed of a Cu matrix reinforced with particles of SiC ceramics in the proportion of 60/40 (metal/ceramics), shown in Figure 2, with the average size of particles 30 μm, in the form of discs Ø 15 × 3 mm.

**Figure 3:**
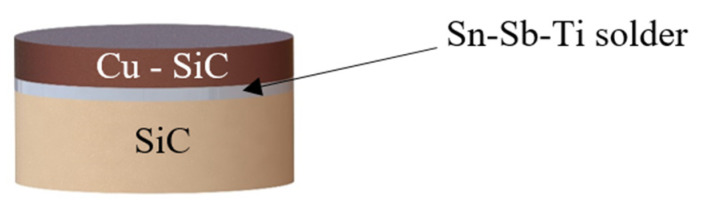
Assembly of a soldered joint for analysis of solder/substrate boundaries.

The scheme of the soldered joint prepared for the chemical analysis of solder/substrate boundaries is shown in Figure 3.

**Figure 4:**
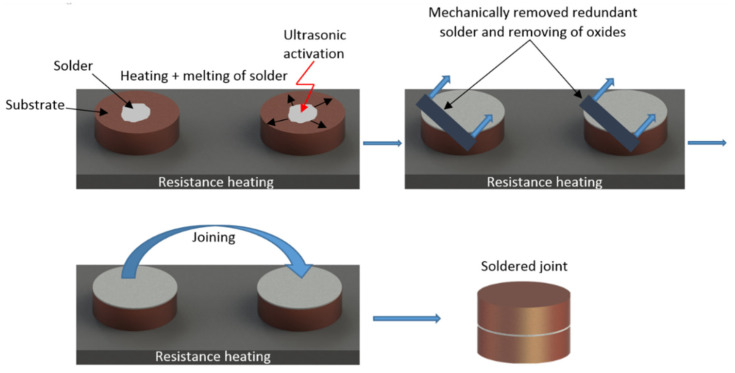
Schematic representation of the soldering process in the presence of ultrasonic power.

The joints were fabricated using a hot plate with thermostatic regulation. The SiC substrate was laid on the hot plate, and the solder heated at the soldering temperature was deposited on it. Soldering was performed using ultrasonic equipment Hanuz UT2 with the parameters given in Table 2. Solder activation was realized via an encapsulated ultrasonic transducer consisting of a piezo-electric oscillating system and a titanium sonotrode with an end tip diameter of 3 mm. The soldering temperature was 260 °C. The soldering temperature was checked by continuous temperature measurement on the hot plate using a NiCr/NiSi thermocouple. The time of ultrasonic power acting was 5 s. Soldering was performed without the use of flux. After ultrasonic activation, the redundant layer of oxides on the molten solder surface was removed. A similar process was also performed on the other substrate. Subsequently, these substrates with molten solder were placed on each other and the joint was formed. We have reported this procedure of soldered joint fabrication in another study [14]. The schematic representation of this process is shown in Figure 4.

Metallographic preparation of specimens from soldered joints was realized by standard metallographic procedures used for specimen preparation. Grinding was performed using SiC emery papers with 240, 320, and 1200 grains/cm^2^ granularity. Polishing was performed with diamond suspensions with grain size 9, 6, and 3 μm. The final polishing was performed using of polishing emulsion OP-S (Struers) with 0.2 μm granularity.

The solder microstructure was studied using scanning electron microscopy (SEM) on microscopes TESCAN VEGA 3 and JEOL 7600 F with X-ray micro-analyzer Microspec WDX-3PC for performing qualitative and semi-quantitative chemical analysis.

For identification of phase composition, X-ray diffraction analysis was applied. This was realized on solder specimens of dimensions 10 × 10 mm by XRD diffractometer PANalytical X’Pert PRO.

DSC analysis of the Sn–Sb–Ti solder was performed on equipment Netzsch STA 409 C/CD in a shielding of Ar gas with 6N purity.

**Figure 5:**
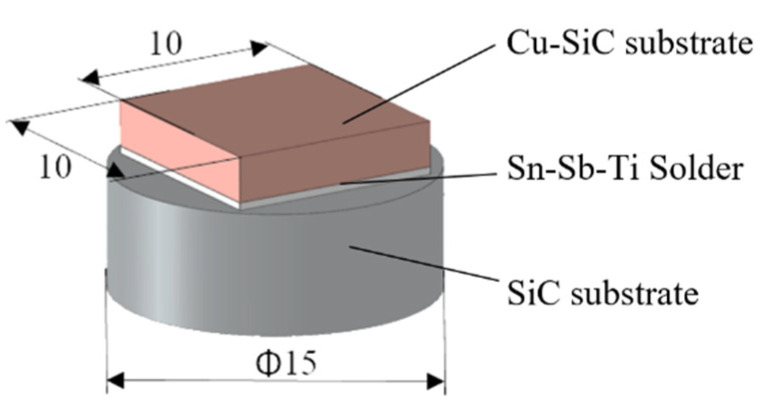
Schematic representation of the specimen for the shear strength test of the Cu–SiC/Sb–Sb–Ti/SiC joint.

**Figure 6:**
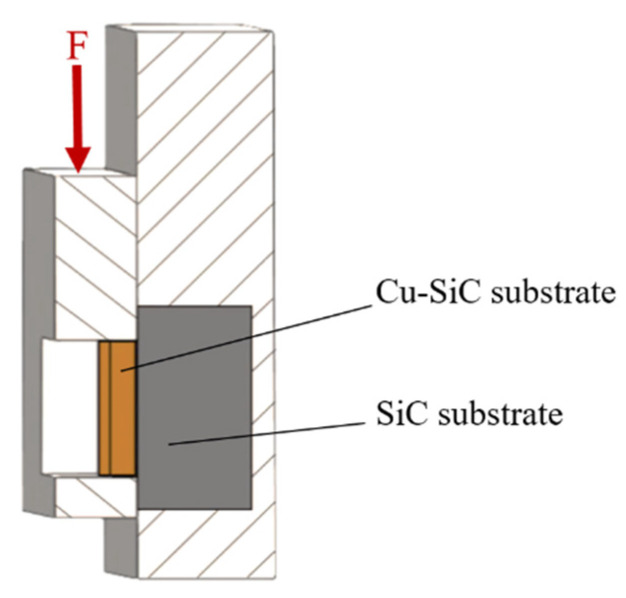
Scheme of shear strength measurement.

For determining mechanical properties of the soldered joints, the shear strength test was performed. The schematic representation of the specimen is shown in Figure 5. The shear strength was measured on the versatile tearing equipment LabTest 5.250SP1-VM. For the change in direction of tensile force, a jig with the defined shape of the test specimen was applied (Figure 6). The shearing jig ensures a uniform shear loading of the specimen in the plane of the solder and substrate boundary.

## 3. Results

### 3.1. Differential Thermal Analyses (DTA)

**Figure 7:**
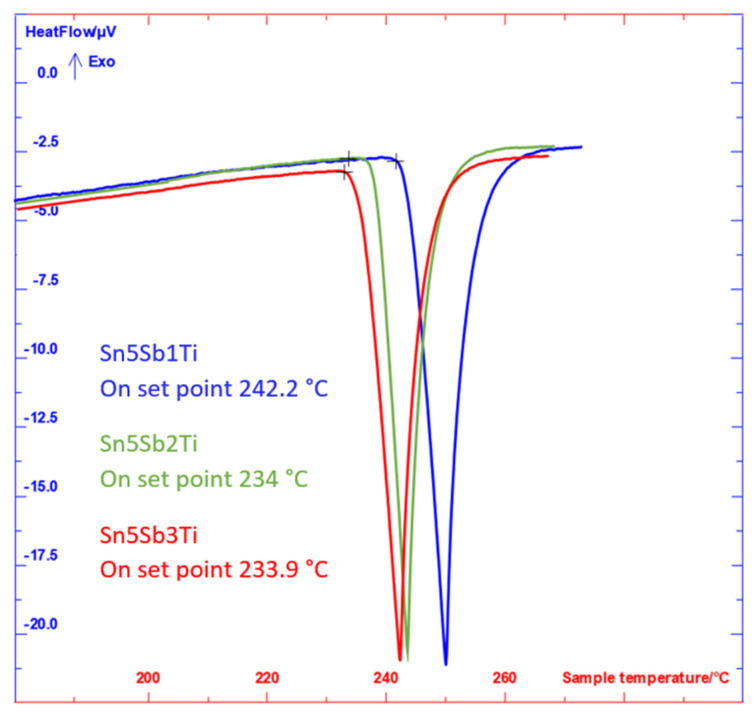
DTA analysis of specimens of soldering alloys of the type SnSb5TiX (2nd measurement).

From the analysis of records in Figure 7, only one reaction was detected in the range of temperatures below 280 °C, namely a peritectic one. Table 3 gives the onset values measured on heating and also on cooling down.

Table 3 suggests that a higher titanium content shifts the peritectic reaction toward lower temperatures. On the contrary, a higher Sb content shifts the transformation temperature toward higher values.

**Figure 8:**
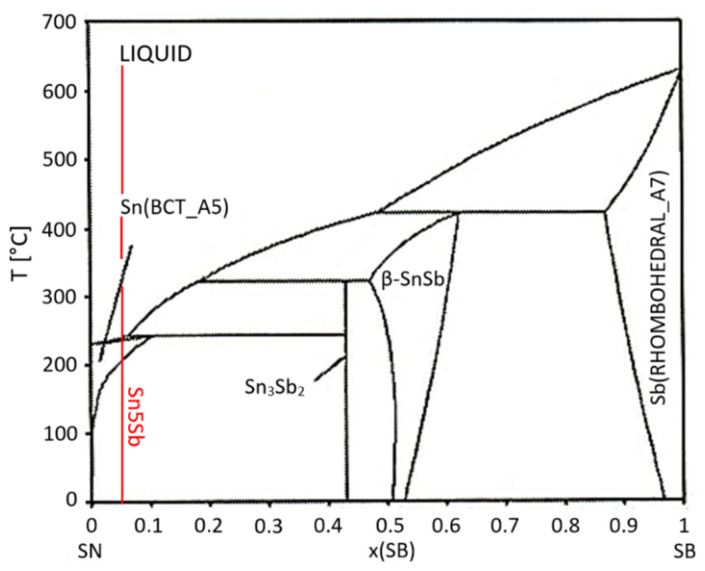
Binary system of tin–antimony [38]. The red line indicates the chemical composition of the Sn5Sb base solder.

Sinn-Wen et al. [2] reported that the peritectic reaction takes place in the binary system of Sn-Sb (Figure 8) at the temperature of 243 °C, which corresponds well with our results:

L + Sn_3_Sb_2_ = (Sn),

where L is for liquid and Sn is for the solid solution of Sn.

**Figure 9:**
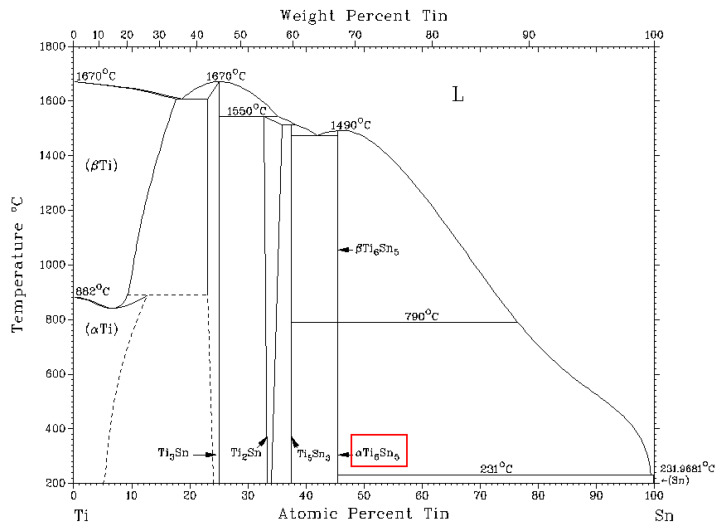
Equilibrium binary diagram of the tin-titanium system [39]. The red box indicates the phase identified in the solder structure.

It is probable that other peaks will appear at considerably higher temperatures, when primary precipitation of dendrites rich in titanium occurs, as follows from the binary diagram of Ti–Sn (Figure 9).

The difference in values of the onset point on heating and cooling down may be attributed to the heterogeneity of the initial alloy, owing to precipitated phases with high a titanium content, i.e., Ti_6_(Sn,Sb)_5_, leading to exhaustion of a considerable titanium portion, when the remaining titanium undergoes a secondary reaction with the Sn–Sb melt, forming the brittle TiSnSb phase.

The DTA/TG analysis of the studied alloy was performed twice, at the heating and cooling rates of 5 °C/min. The results concerning heating are presented in Figure 7. From the results of DTA analysis, only one significant phase reaction with a pronounced thermal effect was observed. The onset point on double heating corresponded to temperatures of 227.8 and 225.9 °C and on cooling down corresponded to temperatures of 224.6 and 224.1 °C. The peak on heating corresponded to temperatures of 240.2 and 241.6 °C. The DTA analysis did not record any other reactions. The difference in values of the onset point on heating was caused by heterogeneity of the initial alloy, owing to precipitated phases, namely the primary solidified phase of acicular morphology with a high titanium content, i.e., Ti_6_(Sn,Sb)_5_, leading to exhaustion of a considerable portion of titanium, with the remaining titanium reacting with the Sn–Sb melt to form the brittle TiSnSb phase. This fact was also proved by the structural and SEM/EDX analyses.

### 3.2. Microstructure of the Sn5Sb3Ti Solder

**Figure 10:**
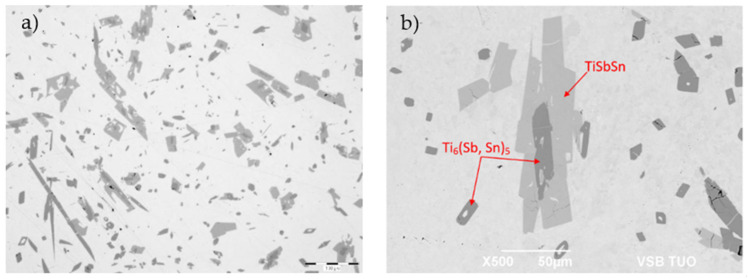
Microstructure of the Sn5Sb3Ti phase; (**a**) from an optical microscope in the as-etched condition; (**b**) from SEM in BSE mode at a higher magnification.

The microstructure of the soldering alloy of the type Sn5Sb3Ti (Figure 10) is formed of the solid solution (Sn) + Sb_3_Sn_2_ phase. The solder matrix contains non-uniformly distributed intermetallic phases of titanium, antimony, and tin. The microstructure with the designation of phases is documented in Figure 10b. Microhardness measurements of individual phases are documented in Table 4.

**Figure 11:**
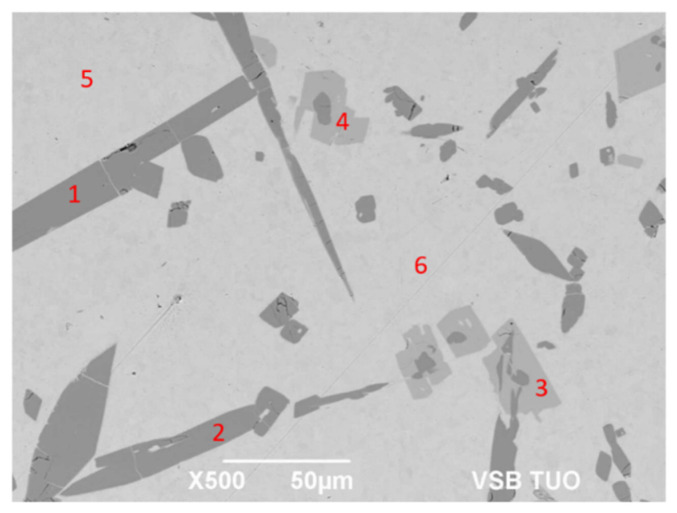
Representation of measuring points of the Sn5Sb3Ti solder.

For determination of the chemical composition of individual components of the soldering alloy, EDX analysis was performed (Table 5). The points of measurement are shown in Figure 11, designated with numbers 1 to 6.

The microstructure (Figure 11) has revealed different morphologically and chemically diverse constituents:

The matrix (Spectra 5 and 6) consists of a mixture with the average content of 97.5 wt.% Sn and around 2.5 wt.% Sb. Titanium was not observed in the matrix. The majority planar proportion in the matrix falls on tin and the minority one on the Sb_3_Sn_2_ phase (Figure 6). The solid solution of tin shows just a limited solubility of antimony.

The bright-gray phase (Spectra 3 and 4) contains all three elements: Ti (16.5 wt.%), Sn (50 wt.%), and Sb (33.5 wt.%). The proportions of atoms stoichiometrically correspond to the composition formula of the TiSbSn phase.

The dark-gray phase with an acicular structure (Spectra 1 and 2) also contains all three elements: Ti (35.5 wt.%), Sn (33 wt.%), and Sb (31.5 wt.%). Regarding the mutual proportions of atoms, the composition of this phase stoichiometrically corresponds to the formula of Ti_6_(Sb,Sn)_5_.

**Figure 12:**
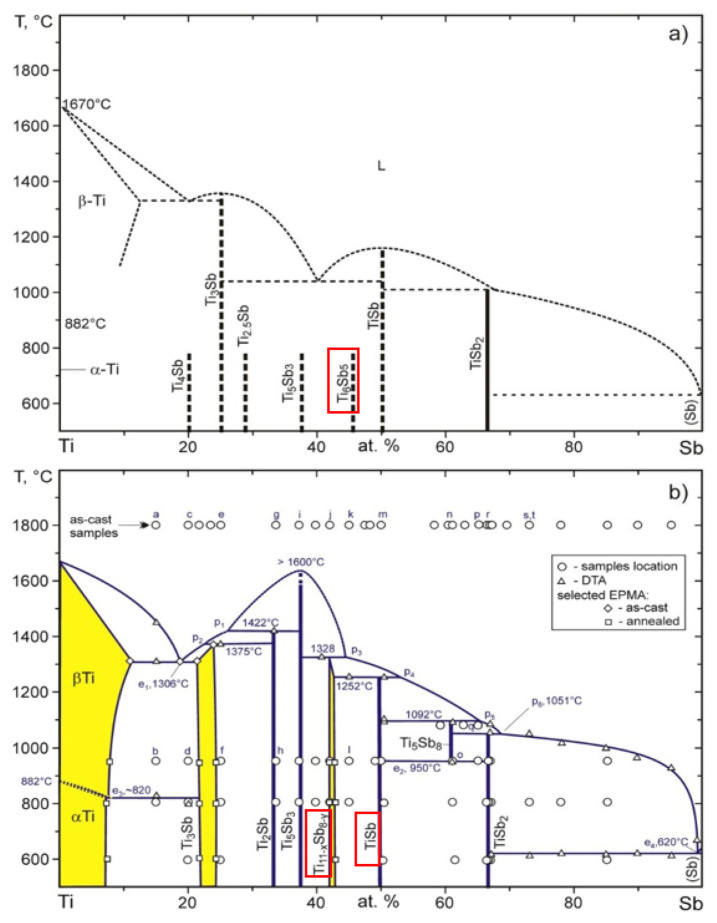
Equilibrium diagram of titanium—antimony; (**a**) partial [40]; (**b**) completed [40].

It is probable that Sn and Sb mutually substitute each other in both these phases. The ternary diagram of Sb–Sn–Ti [29] reveals the phase compositions at temperatures of 600, 800, and 1000 °C. At a soldering temperature of 260 °C, these diagrams are not available. Therefore, we present the binary diagrams in Figure 9 and Figure 12. Regarding the binary diagram of Ti–Sn, the formation of the Ti_6_Sn_5_ phase seems to be probable.

From the results of metallography and SEM/EDX analysis, the primary reaction of titanium with the tin melt, containing antimony addition, may be supposed. The acicular constituents of the Ti_6_(Sb,Sn)_5_ phase with a high titanium content were formed initially This phase then reacted with the tin melt to form small islands of irregular, mostly sharp-edged shape, composed of the TiSbSn phase. This phase was also confirmed by the ternary diagram in the study by Berger et al. [29].

In the case of the Sn–Sb system, a peritectic type of diagram is seen on the tin side when the reaction takes place at the temperature of 243 °C and the composition of liquidus corresponds to 6.5 at.% Sb (Figure 8).

In the Sn–Ti system, a monotectic type of diagram is seen, where the eutectic reaction takes place at the temperature around 231 °C and the Ti_6_Sn_5_ phase is formed. In the case of the Sb–Ti system, the formation of Ti_11−x_Sb_8−y_ (resp. Ti_6_Sb_5_) phases is probable (Figure 12). These phases were also confirmed by a ternary diagram in the study [29].

Regarding the studied alloy of the type Sn5Sb3Ti, in accordance with the binary diagram of Sn–Sb, we are slightly to the left from the peritectic point (Figure 8), the same as in the case of the liquidus composition at the peritectic reaction. The solubility of Sb in Sn at equilibrium conditions decreases with decreasing temperature.

**Figure 13:**
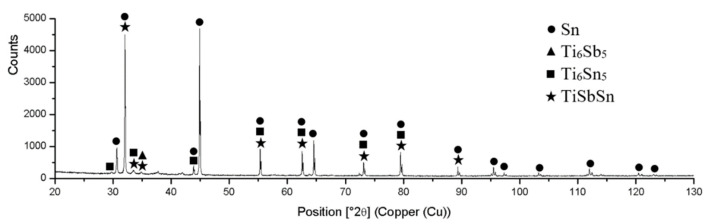
XRD analysis of the Sn5Sb3Ti solder.

The XRD analysis of the Sn5Sb3Ti solder proved the presence of Sn and Sb and also the presence of intermetallic phases of titanium and antimony, namely Ti_6_Sb_5_, Ti_6_Sn_5_, and TiSbSn, which were confirmed by the ternary diagram from the study [29]. The record from diffraction analysis is documented in Figure 13.

**Figure 14:**
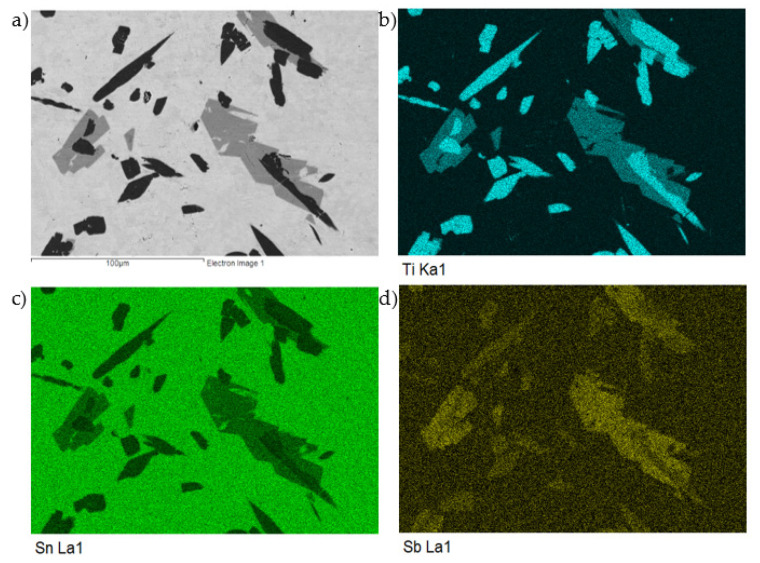
The map of Ti, Sn, and Sb elements in the microstructure of the SnSb5Ti3 solder. SEM photo of (**a**) the microstructure, (**b**) Ti, (**c**) Sn, and (**d**) Sb.

The planar distribution of titanium and antimony phases Ti_6_(Sb,Sn)_5_ and TiSbSn in the tin matrix with a low Sb content is documented in Figure 14.

### 3.3. Tensile Strength of Soldering Alloys

The mechanical tests were oriented to determine the effect of a small Ti addition on the tensile strength of soldering alloy of the type Sn5Sb. Three compositions of soldering alloy containing 1, 2, and 3 wt.% of Ti were used.

**Figure 15:**
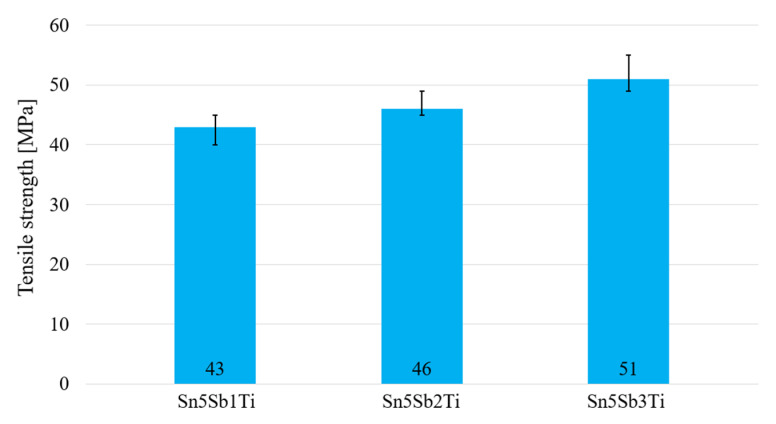
Tensile strength of soldering alloys of the type Sn5Sb1-3Ti.

The dimensions of test pieces (Figure 1) were proposed and calculated. For tensile strength measurement of soldering alloys, three specimens of each experimental soldering alloy were used. The loading rate of the specimen was 1 mm/min. The results of the tensile test are documented in the graph in Figure 15.

The alloy containing 1 wt.% of Ti exhibits the lowest tensile strength, namely 43 MPa. The strength of the solder increases with increasing Ti content. The highest tensile strength was achieved with the solder containing 3 wt.% of Ti (51 MPa). The mentioned facts suggest that titanium addition to an active solder partially increases the tensile strength of soldering alloys of the type Sn–Sb–Ti, since it reacts with antimony to form the intermetallic phases Ti_6_(Sb,Sn)_5_ and TiSbSn, which reinforce the tin matrix of the solder.

### 3.4. Microstructure of SiC/Sn5Sb3Ti/Cu–SiC Joint

**Figure 16:**
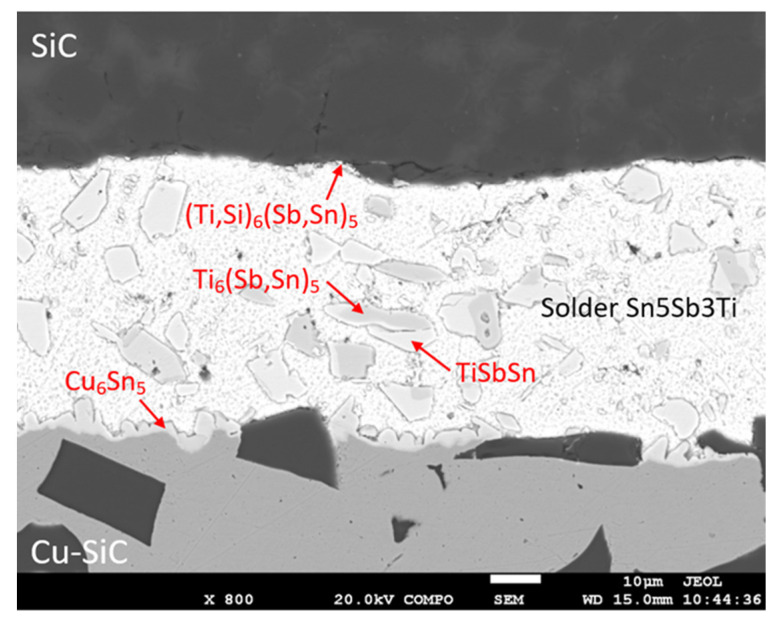
Microstructure of the SiC/Sn5Sb3Ti/Cu–SiC joint from the SEM analysis in BSE mode.

The soldered joint of SiC/Sn5Sb3Ti/Cu–SiC was fabricated at the temperature of 260 °C. Owing to ultrasound activation, an acceptable joint was achieved in the soldering process, which did not contain any cracks, nor inhomogeneities. The microstructure of the soldered joint is shown in Figure 16.

Figure 16 shows that large particles of titanium, tin, and antimony phases occur in the solder matrix. A continuous transition zone with the occurrence of new intermetallic phases was formed in the boundary with the composite material of Cu–SiC.

**Figure 17:**
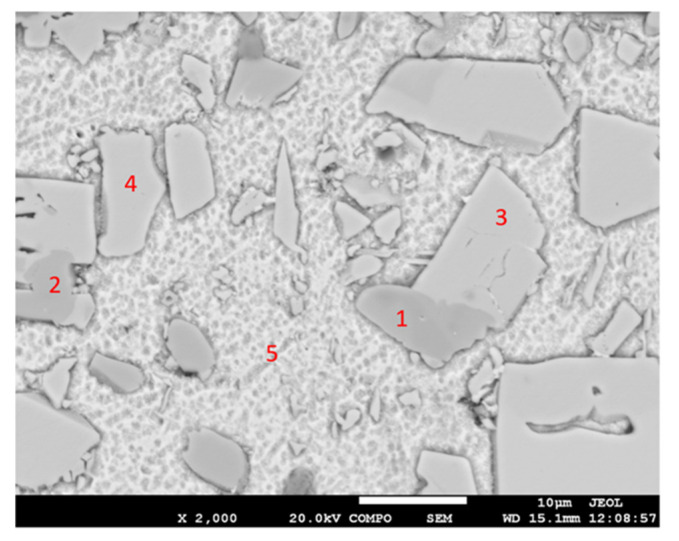
Representation of measuring points of the SiC/Sn5Sb3Ti/Cu–SiC joint.

For determining the chemical composition and identifying individual phases, EDX analysis of the soldered joint was performed (Figure 17 and Table 6).

The measurement was performed at five points, namely Spectra 1 to 5 (Figure 17). Spectra 1 and 2 represent the gray phase, with a high Ti content (~35 wt.% Ti). It corresponds to the composition of the Ti_6_(Sb,Sn)_5_ phase. It is probable that Sn and Sb mutually substitute in both phases.

The Spectra 3 to 4 represent the bright-gray phase, with a lower Ti content (~16 to 17 wt.%). The composition of this phase corresponds to the TiSbSn phase. Spectrum 5 represents the solid solution of tin.

### 3.5. Analysis of the Transition Zone in the SiC/Sn5Sb3Ti Joint

**Figure 18:**
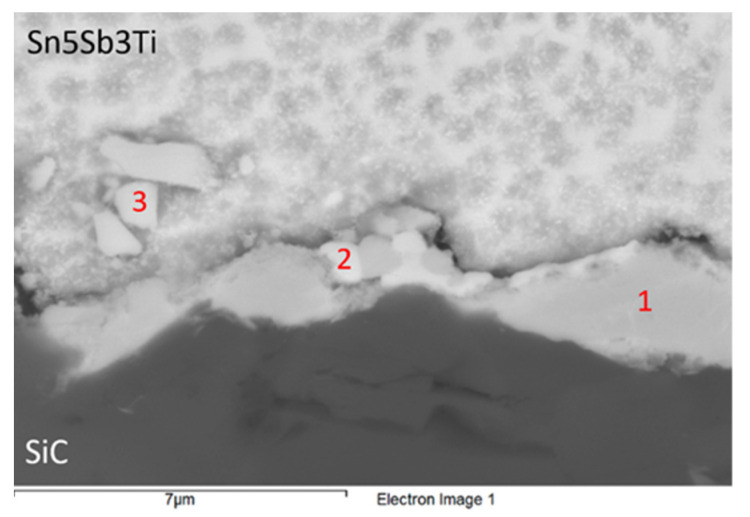
Representation of measuring points on the SiC/Sn5Sb3Ti interface.

Based on the previous studies [14,22], it was supposed that the active Ti element will be concentrated in the boundary with the ceramic SiC material, where it will form new phases. The interaction of titanium was observed in the solder/SiC ceramics boundary (Figure 18 and Table 7), whereby a higher amount of Ti was precipitated in this boundary. Spot analysis has proved the presence of titanium in amounts from 17.5 to 33 wt.%. However, the presence of increased antimony content, from 12 to 30 wt.%, was also observed in the reaction layer on the boundary. It is supposed that both these elements contribute to bond formation with the ceramic material of SiC. The presence of ceramics in the reaction layer in amounts from 3 to 7 wt.% proves the mutual interaction between the solder and the substrate.

From the microstructure shown in Figure 18, it is clearly visible that the solder matrix is formed of a peritectic mixture of a solid solution (Sn, bright zone) and Sb_3_Sn_2_ (darker, bead-like constituents). The measurement point Spectrum 1 occurs directly on the SiC/solder boundary. The chemical composition stoichiometrically corresponds to the intermetallic phase (Ti,Si)_6_(Sb,Sn)_5_, where silicon, which has substituted titanium, is partially bound. Thus, it is evident that an interaction between the ceramics and solder took place.

Based on the chemical composition, a small zone of around 1 μm was identified in the measurement point Spectrum 2. A quaternary phase is present in this case. It is interesting that the proportion of atoms (Si + Ti):(Sn + Sb) = 50:50. The substitution of tin and antimony in this phase is again supposed. Regarding the proportion of all elements present in Spectrum 2, the phase type Si_7_Ti_10_(Sb,Sn)_17_ may be of concern. The proportion of atoms Sn:Sb = 21:4. In the measurement point Spectrum 3, a phase in the solder matrix zone occurs but relatively close to the SiC/solder boundary. This zone stoichiometrically corresponds to the chemical composition of the intermetallic phase of TiSbSn. The substitution of Sn and Sb has also occurred here, with a higher proportion of Sn:Sb. In addition, a slight dilution of silicon (around 0.74 wt.%) has occurred, with partially substituted titanium.

**Figure 19:**
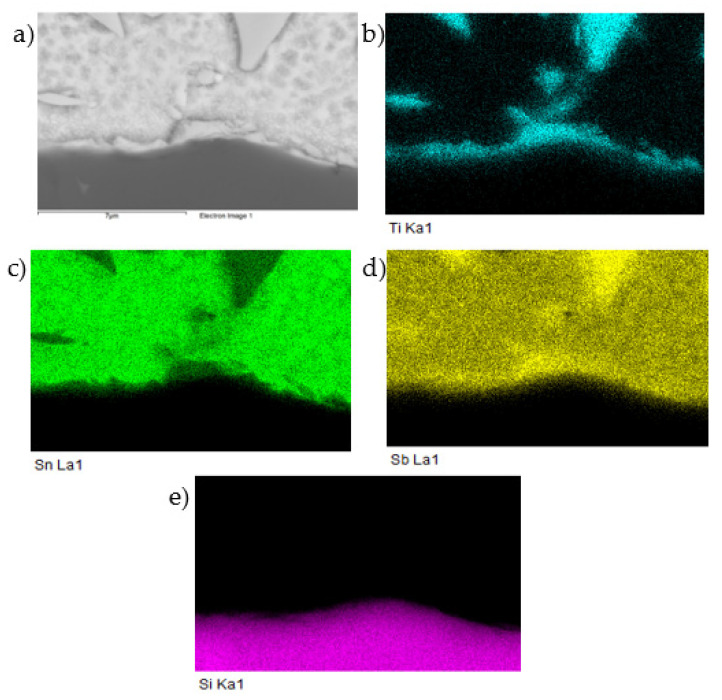
Planar distribution of Ti, Sn, Sb, and Si elements in the boundary of the SiC/Sn5Sb3Ti joint; SEM photo of (**a**) the boundary, (**b**) Ti, (**c**) Sn, (**d**) Sb, and (**e**) Si.

The planar distribution of elements in the boundary is documented in Figure 19. From this distribution, it is obvious that Ti significantly participates in bond formation with SiC ceramics.

**Figure 20:**
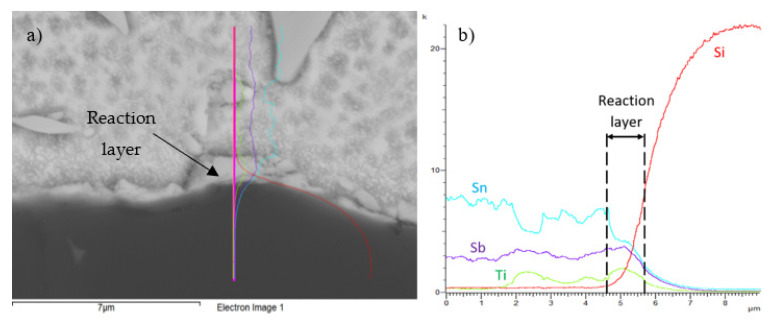
Line EDX analysis of the SiC/Sn5Sb3Ti joint; (**a**) transition zone with a marked line; (**b**) concentration profiles of Sb, Sn, Ti, and Si elements.

The line analysis and concentration profiles of Ti and Sb elements (Figure 20) prove that both these elements precipitate on the boundary with the ceramic material of SiC. The effect of antimony on the formation and bond strength with the ceramic material of Al_2_O_3_ is also proved by the study [41], where the shear strength of the joint was increased by Sb addition to the Sn–Zn–Sb solder.

From the above, the following mechanism of bond formation may be concluded. In the soldering process, titanium and antimony are distributed to the boundary of the SiC ceramic material, where a reaction layer ensuring the wetting of SiC ceramics is formed. Between the active elements and the ceramic material, a reaction takes place forming reaction products, which allow the wetting of ceramics by an active solder. The thickness of the reaction layer is 1 to 3 µm.

### 3.6. Analysis of the Transition Zone in the Cu–SiC/Sn5Sb3Ti Joint

**Figure 21:**
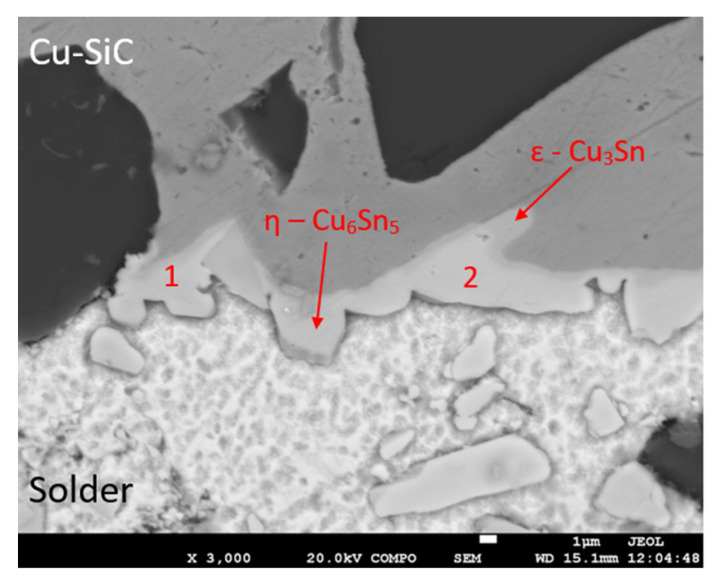
Representation of the boundary in the Cu–SiC/Sn5Sb3Ti joint.

The transition zone in the joint was analyzed. A pronounced transition zone was formed in the boundary of the Cu/Sn5Sb3Ti joint, where the Cu phase was identified, which is the result of the interaction of the copper substrate and the solder (Figure 21). The undulating character of the boundary with the copper substrate is the result of the action of the ultrasound and the molten solder on the surface of the copper substrate. The thickness of the new transition zone with intermetallic phases of copper is 2.5 to 5 µm.

Measurement in this zone was performed at two points, Spectra 1 and 2. The results of the measurement are given in Table 8.

**Figure 22:**
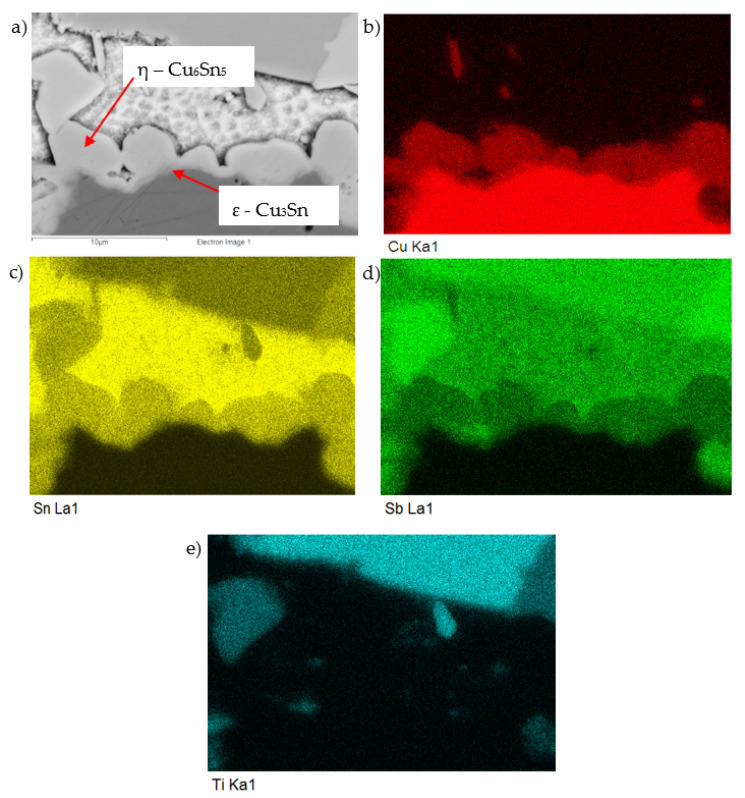
Map of elements in the boundary of the Cu–SiC/Sn5Sb3Ti joint; SEM photo of (**a**) the boundary, (**b**) Cu, (**c**) Sn, (**d**) Sb, and (**e**) Ti.

In the points of measurement Spectra 1 and 2, the η-Cu_6_Sn_5_ phase was clearly identified. The distribution map of elements in the boundary of the Cu–SiC/Sn5Sb3Ti joint is documented in Figure 22a, clearly showing the η-Cu_6_Sn_5_ phase, where the ε-Cu_3_Sn phase is also partially visible.

**Figure 23:**
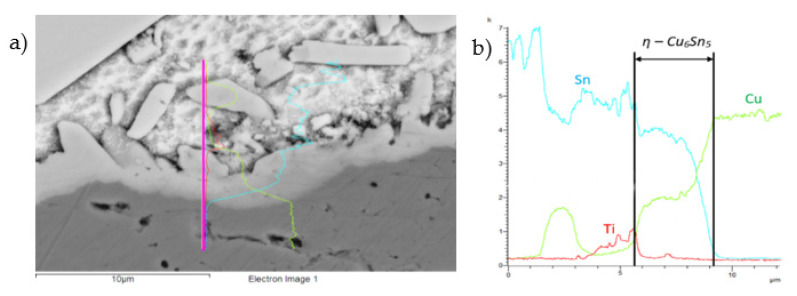
The line EDX analysis of the Cu–SiC/Sn5Sb3Ti joint; (**a**) transition zone with a marked line; (**b**) concentration profiles of Sn, Sb, Ti, and Cu elements.

The concentration profiles of Cu, Sn, Sb, and Ti elements (Figure 23) on the boundary of Cu–SiC/Sn5Sb3Ti joints have revealed the transition zone with the formation of the η-Cu_6_Sn_5_ phase.

### 3.7. Shear Strength of Soldered Joints

**Figure 24:**
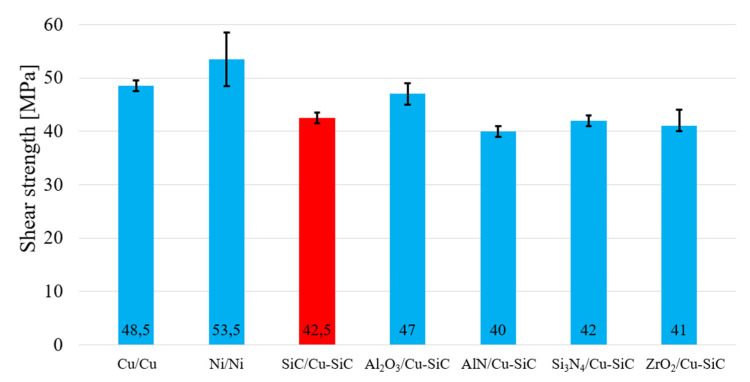
Shear strength of soldered joints fabricated with the Sn5Sb3Ti solder.

The research in this study was primarily oriented to soldering SiC ceramics with a composite Cu–SiC substrate. Due to possibilities of application of the active solder of the type Sn5Sb3Ti and its further introduction into practice, the testing of shear strength was extended to other metallic (Cu and Ni) and ceramic (Al_2_O_3_, AlN, and Si_3_N_4_) materials. Ceramic materials were always tested in combination with the composite substrate of Cu–SiC. Metallic materials were mutually soldered, namely Cu/Cu and Ni/Ni. The measurement was performed on three specimens of each material. The results of the average shear strength of the joints are documented in Figure 24. The highest shear strength, achieved with the ceramic/Cu–SiC composite joint, was observed in the case of the Al_2_O_3_/Cu–SiC joint (47 MPa). In the case of other combinations with ceramic materials such as SiC/CuSiC, Si_3_N_4_/Cu–SiC, and ZrO_2_/Cu–SiC, a comparable average shear strength in the range from 40 to 42.5 MPa was measured. In the case of metallic materials, the average shear strength of two Ni materials was 53.5 MPa. However, in this case, the widest scatter of measurement, ranging from 48 to 59 MPa, was also observed. The average shear strength of Cu/Cu joint is 48.5 MPa. Although in the case of the SiC/Cu–SiC combination of materials, a lower shear strength of the joint was measured (42.5 MPa), the limit criterion for soldering power semiconductors is 40 MPa. The Sn5Sb3Ti solder thus meets this condition for all materials tested.

**Figure 25:**
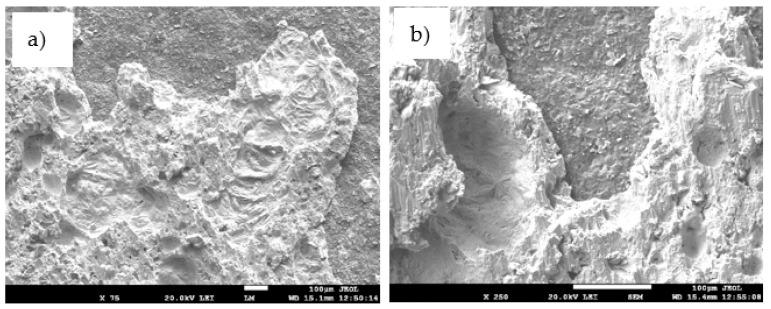
The fractured surface of the soldered joint of SiC/Sn5Sb3Ti/CuSiC; (**a**) magnification 75×; (**b**) magnification 250×.

**Figure 26:**
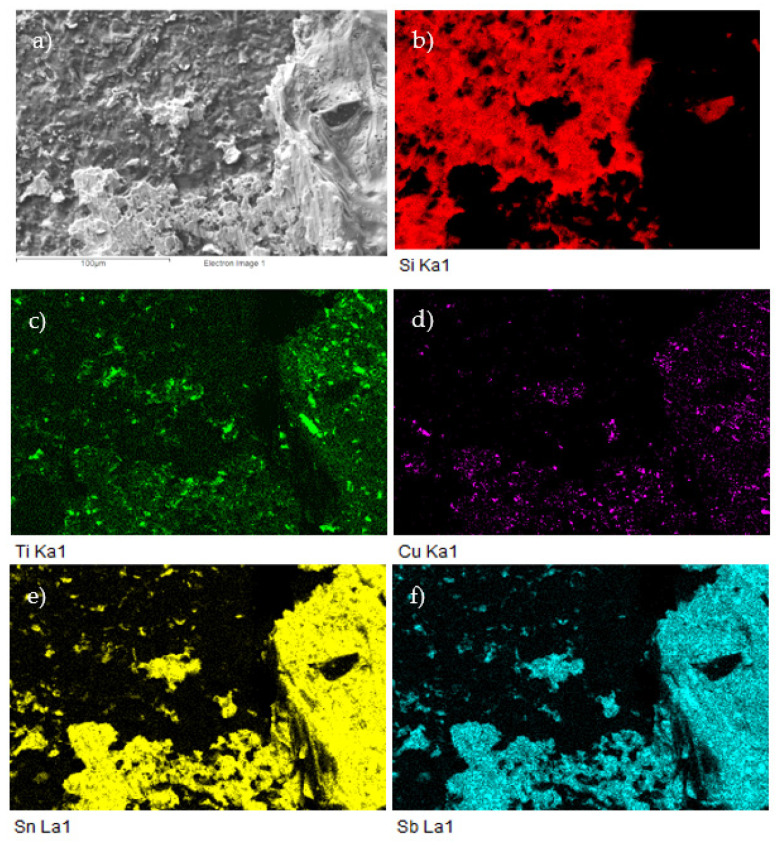
The fractured surface of the soldered joint of SiC/Sn5Sb3Ti/Cu–SiC and the planar distribution of individual elements; (**a**) fracture morphology; (**b**) Si; (**c**) Ti; (**d**) Cu; (**e**) Sn; (**f**) Sb.

For more precise identification of the mechanism of bond formation, the fractured surfaces of joints were analyzed. Figure 25a,b shows the fractured surface on the boundary of the SiC/Sn5Sb3Ti/Cu–SiC joint. It is evident that the fractured surface from the side of SiC ceramics remained partially covered with the solder. Solder coverage was approximately 80%. Ductile fracture was observed in the solder. Planar analysis of the distribution of Si, Ti, Cu, Sn, and Sb elements on the fractured surface was performed, as documented in Figure 26b–f. The planar distribution of Si element shown in Figure 26b represents the SiC ceramics, and local spots (where the solder was pulled out) may be observed. From the distribution of Ti on the fractured surface, shown in Figure 26c, it may be concluded that Ti is bound to SiC ceramics and thus it contributes considerably to bond formation.

**Figure 27:**
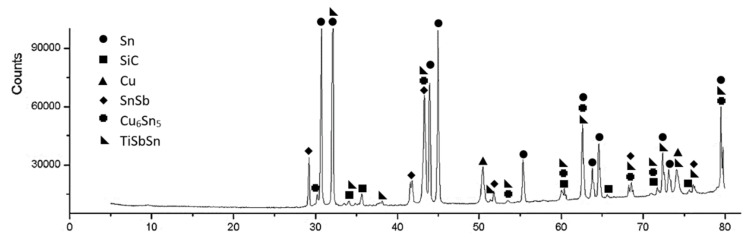
XRD analysis of the boundary in the SiC/Sn5Sb3Ti/CuSiC joint.

XRD analysis was performed in the boundary of the SiC/Sn5Sb3Ti joint (Figure 27). The analysis proved the presence of a titanium phase of the type TiSbSn and a copper phase of the type Cu_6_Sn_5_ on the fractured surface. Moreover, the SnSb phase was also observed, which was not identified by EDX analysis, and its existence is also confirmed by the binary diagram of Sn–Sb shown in Figure 8.

## 4. Conclusions

The aim of the research was to characterize the soldering alloy of the type Sn–Sb–Ti and study whether the proposed composition of the solder would be suitable for soldering SiC ceramics with a metal–ceramic composite of the type Cu–SiC with the application of ultrasonic soldering. The following results were achieved:

- For determining the melting point, DTA analysis was applied. From analysis of the DTA results, only one reaction was identified, namely the peritectic one at an approximate temperature of 243 °C. Sn–Sb alloys containing 1, 2, and 3 wt.% of Ti were assessed. It was found that a higher Ti content shifts the peritectic reaction toward lower temperatures. The addition of Ti content lowered the melting point of the Sn5Sb3Ti alloy, which resulted in a faster transition from solid to liquid.
- The solder structure consisted of a tin matrix, which contained the solid solution (Sn) + Sb_3_Sn_2_ phase. The solder matrix contained non-uniformly distributed intermetallic phases of titanium, antimony, and tin. In addition, the formation of acicular constituents of the Ti_6_(Sb,Sn)_5_ phase with a high content of titanium primarily occurred. This secondary phase reacted with the tin melt to form islands of an irregular, mostly sharp-edged shape, namely the TiSbSn phase. The formation of titanium-containing intermetallic phases resulted in the strengthening of the tin matrix of the solder.
- The Sn5Sb-based soldering alloy attained the average tensile strength of 43 to 51 MPa depending on the titanium content. It was found that Ti addition to a solder partially increases the tensile strength of soldering alloys of the type Sn5Sb. This results from solder matrix strengthening due to intermetallic phases of titanium.
- The SiC/solder bond was formed as follows: During the soldering process, titanium and antimony were distributed to the boundary with the ceramic SiC material, where a reaction layer, ensuring the wettability of SiC ceramics, was formed. Between the active element and the ceramic material, a reaction took place forming reaction products that wet the ceramic due to an active solder. The reaction product, the (Ti,Si)_6_(Sb,Sn)_5_ intermetallic phase, was identified, where silicon, which has substituted titanium, was partially bound. This suggests that interaction between the ceramics and the solder took place.
- A transition zone was formed on the boundary of the Cu–SiC/solder joint, whereby dilution of Cu from the metal–ceramic composite Cu–SiC occurred in the liquid tin solder. Two phases were identified: the wettable η-Cu_6_Sn_5_ phase, in contact with the solder, and the non-wettable ε-Cu_3_Sn phase, in contact with the copper composite.
- The measurements of shear strength were performed on a wide range of metallic and ceramic materials. The average shear strength of a combined joint of SiC/Cu–SiC, fabricated with the Sn5Sb3Ti solder was 42.5 MPa. The limit criterion for soldering power semiconductors is the shear strength of 40 MPa. The Sn5Sb3Ti solder meets this condition in the case of all materials studied.

The Sn–Sb–Ti solder is a direct competitor of the S-Bond active solder. The production of solder is cheaper, and the presence of antimony increases its strength. The application temperatures range is also wider.

## Figures and Tables

**Table 1:** Composition of the Sn–Sb–Ti alloy and the results of chemical analysis by the ICP-AES method (wt.%).

Specimen	Charge (wt.%)			ICP-AES (wt.%)		
	Sn	Sb	Ti	Sn	Sb	Ti
Sn5Sb1Ti	94.0	5.0	1.0	Balance	4.98 ± 0.15	1.21 ± 0.25
Sn5Sb2Ti	93.0	5.0	2.0	Balance	3.94 ± 0.48	1.79 ± 0.10
Sn5Sb3Ti	92.0	5.0	3.0	Balance	5.18 ± 0.26	3.31 ± 0.34

**Table 2:** Ultrasonic soldering parameters.

Ultrasound power	400	(W)
Working frequency	40	(kHz)
Amplitude	2	(μm)
Soldering temperature	260	(°C)
Time of ultrasound activation	5	(s)

**Table 3:** The values of phase transformation in Sn5SbXTi solders measured by DTA analysis.

Composition	Onset Point on Heating (°C)		Onset Point on Cooling Down (°C)	
	First measurement	Second measurement	First measurement	Second measurement
Sn5Sb1Ti	247.5	242.2	239.6	239.6
Sn5Sb2Ti	241.8	234.0	230.7	230.9
Sn5Sb3Ti	234.9	233.9	228.6	228.3

**Table 4:** HV microhardness of individual components of the Sn5Sb3Ti solder.

	Sn Matrix	Bright-Gray TiSbSn Phase	Dark-Gray Ti_6_(Sb,Sn)_5_ Phase
HV 0.01	20	174	939

**Table 5:** EDX analysis of the Sn5Sb3Ti solder.

Spectrum	Sn (wt.%)	Sb (wt.%)	Ti (wt.%)	Solder Component
Spectrum 1	32.5	31.7	35.8	Ti_6_(Sb,Sn)_5_ phase
Spectrum 2	33.9	30.9	35.2	Ti_6_(Sb,Sn)_5_ phase
Spectrum 3	49.6	34.0	16.5	TiSbSn phase
Spectrum 4	50.8	32.8	16.4	TiSbSn phase
Spectrum 5	98.1	1.9	0	Sn + Sb_3_Sn_2_ phase
Spectrum 6	97.0	3.0	0	Sn + Sb_3_Sn_2_ phase

**Table 6:** Point EDX analysis of the SiC/SnSb5Ti3/Cu–SiC joint.

Spectrum	Sn (wt.%)	Sb (wt.%)	Ti (wt.%)	Solder Component
Spectrum 1	34.7	30.0	35.4	Ti_6_(Sb,Sn)_5_ phase
Spectrum 2	34.6	30.1	35.3	Ti_6_(Sb,Sn)_5_ phase
Spectrum 3	47.4	35.7	16.9	TiSbSn phase
Spectrum 4	46.9	36.8	16.4	TiSbSn phase
Spectrum 5	98.5	1.5	0	Sn + Sb_3_Sn_2_ phase

**Table 7:** Point EDX analysis of the boundary in the SiC/Sn5Sb3Ti joint.

Spectrum	Sn (wt.%)	Sb (wt.%)	Ti (wt.%)	Si (wt. %)	Solder Component
Spectrum 1	33.60	29.99	33.38	3.03	(Ti,Si)_6_(Sb,Sn)_5_ phase
Spectrum 2	63.07	12.16	17.55	7.21	Si_7_Ti_10_(Sb,Sn)_17_ phase
Spectrum 3	61.76	22.81	14.69	0.74	TiSbSn phase

**Table 8:** Point EDX analysis of the boundary in the Cu–SiC/Sn5Sb3Ti joint.

Spectrum	Sn (wt.%)	Cu (wt.%)	Solder Component
Spectrum 1	39.7	60.3	η-Cu_6_Sn_5_
Spectrum 2	39.1	61.0	η-Cu_6_Sn_5_
